# Differential Effects of Cystathionine-γ-lyase–Dependent Vasodilatory H_2_S in Periadventitial Vasoregulation of Rat and Mouse Aortas

**DOI:** 10.1371/journal.pone.0041951

**Published:** 2012-08-03

**Authors:** Carolin Köhn, Johanna Schleifenbaum, István András Szijártó, Lajos Markó, Galyna Dubrovska, Yu Huang, Maik Gollasch

**Affiliations:** 1 Medical Clinic for Nephrology and Internal Intensive Care, Charité Campus Virchow Klinikum and Experimental and Clinical Research Center (ECRC), Max Delbrück Center for Molecular Medicine, Berlin, Germany; 2 Institute of Vascular Medicine, Li Ka Shing Institute of Health Sciences, School of Biomedical Sciences, Chinese University of Hong Kong, Shatin, Hong Kong, China; Medical School of Hannover, United States of America

## Abstract

**Background:**

Hydrogen sulfide (H_2_S) is a potent vasodilator. However, the complex mechanisms of vasoregulation by H_2_S are not fully understood. We tested the hypotheses that (1) H_2_S exerts vasodilatory effects by opening KCNQ-type voltage-dependent (K_v_) K^+^ channels and (2) that H_2_S-producing cystathionine-γ-lyase (CSE) in perivascular adipose tissue plays a major role in this pathway.

**Methodology/Principal Findings:**

Wire myography of rat and mouse aortas was used. NaHS and 5-(4-hydroxyphenyl)-3H-1,2-dithiole-3-thione (ADTOH) were used as H_2_S donors. KCNQ-type K_v_ channels were blocked by XE991. 4-Propargylglycine (PPG) and ß-cyano-l-alanine (BCA), or 2-(aminooxy)-acetic acid (AOAA) were used as inhibitors of CSE or cystathionine-ß-synthase (CBS), respectively. NaHS and ADTOH produced strong vasorelaxation in rat and mouse aortas, which were abolished by KCNQ channel inhibition with XE991. Perivascular adipose tissue (PVAT) exerted an anticontractile effect in these arteries. CSE inhibition by PPG and BCA reduced this effect in aortas from rats but not from mice. CBS inhibition with AOAA did not inhibit the anticontractile effects of PVAT. XE991, however, almost completely suppressed the anticontractile effects of PVAT in both species. Exogenous l-cysteine, substrate for the endogenous production of H_2_S, induced vasorelaxation only at concentrations >5 mmol/l, an effect unchanged by CSE inhibition.

**Conclusions/Signficance:**

Our results demonstrate potent vasorelaxant effects of H_2_S donors in large arteries of both rats and mice, in which XE991-sensitive KCNQ-type channel opening play a pivotal role. CSE-H_2_S seems to modulate the effect of adipocyte-derived relaxing factor in rat but not in mouse aorta. The present study provides novel insight into the interaction of CSE-H_2_S and perivascular adipose tissue. Furthermore, with additional technical advances, a future clinical approach targeting vascular H_2_S/KCNQ pathways to influence states of vascular dysfunction may be possible.

## Introduction

Hydrogen sulfide (H_2_S), next to nitric oxide (NO) and carbon monoxide (CO), is the third gasotransmitter described to exert vasoactive effects on systemic arteries. *In vivo*, H_2_S is mainly produced by cystathionine-γ-lyase (CSE) and cystathionine-ß-synthase (CBS), using l-cysteine as the substrate [1], [2]. CSE is the main source of endogenous H_2_S in the vascular wall [1]. However, additional pathways that produce H_2_S seem to exist in red blood cells [3], which may potentially affect vascular tone. 3-mercaptopyruvate-sulfurtransferase (3MST) has recently been identified to produce H_2_S in the endothelium and regulate vascular tone [4]. Deficiency of CSE leads to arterial hypertension in mice [5]. H_2_S exerts anti-hypertensive effects by vasorelaxation which involves opening of ATP-dependent (K_ATP_) potassium channels, intermediate conductance (IK_Ca_) and small conductance (SK_Ca_) potassium channels [5], [6], suppression of vascular inflammation [7] and/or increase of renin release [8]. Hypoxia can produce excessive production of H_2_S [9]. H_2_S is involved in trauma and circulatory shock [1] and progression of cardiac hypertrophy by attenuating fibrosis [10].

Exogenous H_2_S relaxes a number of blood vessels in different species. Several pathways has been identified to explain its actions, including activation of protein kinase A (PKA) *via* cyclic adenosine-mono-phosphate (cAMP) [11], [12], [13], release of endogenous vasodilator prostanoids [14], activation of K_ATP_ channels [15], and activation of the Cl^−/^HCO_3_^−^ transporter [16]. Low concentrations of H_2_S (<100 µmol/l) can promote vasoconstriction *via* inhibition of the cAMP/PKA pathway [11] and possibly binding of NO by forming nitrosothiols [17]. H_2_S activates phosphatidylinositol-3-kinase (PI3K) and mitogen activated protein kinases (MAPK) [15], [18].

Perivascular adipose tissue (PVAT) reduces the contractile force of isolated rat aortas in response to norepinephrine [19] and other vasoconstrictors in a paracrine manner [20], [21]. Bioassay experiments showed that PVAT secretes an “adipocyte derived relaxing factor” (ADRF) that exerts its anti-contractile effect on systemic peripheral arteries *via* activation of potassium channels in vascular smooth muscle cells [20], [22]. The anti-contractile effect of PVAT has been observed in both large and small vessels in rats, mice, pigs, and humans [23], [24].

Although the nature of ADRF is unknown, it is seemingly a transferable factor that induces opening of K_v_ channels and, thus, leads to hyperpolarization of vascular smooth muscle cells [20], [21]. XE991-sensitive K_v_ channels have been suggested to play an important role in rat aorta [25]. In contrast, K_ATP_ channels are not involved [26]. However, activation of maxi calcium-activated (BK_Ca_) channels has been reported in other studies [27], [28], [29], suggesting possible vessel and species differences. ADRF release and action are independent of the endothelium [20]. A number of factors are discussed as putative ADRF candidates, including Ang 1–7 [27], free radicals or cytokines [30], [31], and methyl palmitate [32]. Recently, our group and Fang et al proposed that CSE-derived H_2_S acts as ADRF in rats [25], [33]. CSE is expressed in peri-aortic adipose tissue in rats and can endogenously produce H_2_S [34], [35]. However, the data of Fang et al. and Schleifenbaum et al. can also be interpreted as CSE-H_2_S rather being modulator of ADRF than primary ADRF. Of note, the CSE/H_2_S/KCNQ pathway has only been investigated in rats, but not studied in different vessels and species. Such studies may reveal its significance as putative common pathway for the anti-contractile effect of perivascular fat.

To investigate the interactions and potential target molecules of ADRF, we studied the differential effects of CSE/H_2_S and H_2_S/KCNQ pathways in the periadventitial vasoregulation of rat and mouse aortas. We tested the hypotheses that (1) H_2_S is a potent vasodilator by opening smooth muscle KCNQ-type K_v_ channels, and that (2) endogenous H_2_S derived from CSE in perivascular adipose tissue is a common mechanism to produce vasorelaxation by opening of these K_v_ channels in rat and mouse aortas.

## Methods

### Isometric Contractions of Rat Vessels

The local animal review board of Berlin-Brandenburg approved all studies, according American Physiological Society criteria. Male Sprague-Dawley rats (200–300 g, 6–8 weeks; Charles River, Sulzfeld/Berlin, Germany) were killed under isoflurane. The thoracic aortas were removed, quickly transferred to cold (4°C), oxygenated (95% O_2_/5% CO_2_) physiological salt solution (PSS), and dissected into 2 mm rings. Perivascular fat and connective tissue were either removed ((−) Fat) or left intact ((+) Fat). After one hour of equilibration, contractile force was measured isometrically using a Schuler Tissue Bath System (Hugo Sachs, Freiburg, Germany) and using standard bath solutions as described [20], [21], [22], [36]. The effects of 4-propargylglycine (PPG) and XE991 on rat aortic rings were studied using both Schuler and Mulvany myograph systems (see below). The basal tone was continuously monitored and adjusted to 1 g in the experiments. Data recording and output was realized using Chart5 for windows (AD Instruments, Spechbach, Germany). The rings were precontracted and equilibrated until a stable resting tension was acquired. Tension is expressed as a percentage of the steady state tension (100%) obtained with isotonic external 60 mmol/l KCl. Vessels were precontracted with serotonin. If not indicated otherwise, drugs were added to the bath solution.

### Isometric Contractions of Mouse Vessels

Male wild-type C57BL/6 mice (25–30 g, 8–12 weeks) were killed under isoflurane or carbon dioxide. The thoracic aortas were removed, quickly transferred to cold (4°C), oxygenated (95% O_2_/5% CO_2_) PSS, and dissected into 2 mm rings whereby perivascular fat and connective tissue were either removed ((−) Fat) or left intact ((+) Fat) as previously described by Löhn et al. Each ring was positioned between two stainless steel wires (diameter 0.0394 mm) in a 5 ml organ bath of a Mulvany Small Vessel Myograph (DMT 610 M; Danish Myo Technology, Denmark) [37]. The organ bath was filled with PSS. The bath solution was continuously oxygenated with a gas mixture of 95% O_2_ and 5% CO_2_, and kept at 37°C (pH 7.4). The rings were placed under tension of 0.3 g [37]. The software Chart5 (AD Instruments Ltd. Spechbach, Germany) was used for data acquisition and display. The rings were precontracted with 60 mmol/l KCl and equilibrated until a stable resting tension was acquired. Tension is expressed as tension/length (mN/mm). If not otherwise indicated vessels were precontracted with serotonin. As in isometric measurements of rat aorta, drugs were added to the bath solution if not indicated otherwise.

### Western Blots

Freshly prepared aortas from mouse or rat were lysed with RIPA buffer (Sigma, Munich, Germany) supplemented with Complete protease inhibitor (Roche) and were homogenized using a Precellys 24 homogenizer (Peqlab, Erlangen, Germany). Fifty microgram protein samples were separated by 12% SDS-PAGE. After semi-dry transfer, non-specific binding sites of the nitrocellulose membrane were blocked with 5% non-fat milk in Tris buffer saline containing 0.1% Tween (TBST). After that the membrane was incubated with the primary antibody (anti-CSE, 1∶200, Sigma). Secondary antibody (anti-β-actin, 1∶5.000, Cell Signaling Technology, Frankfurt/M., Germany) was applied after stripping the membrane. Images were acquired by Odyssey infrared imaging system (LI-COR Biosciences, Bad Homburg, Germany). ß-Actin was used as loading control.

### Materials and Statistics

The composition of PSS (in mmol/l) was 119 NaCl, 4.7 KCl, 1.2 KH_2_PO_4_, 25 NaHCO_3_, 1.2 Mg_2_SO_4_, 11.1 glucose, and 1.6 CaCl_2_. The composition of 60 mmol/l KCl (in mmol/l) was 119 NaCl, 60 KCl, 1.2 KH_2_PO_4_, 25 NaHCO_3_, 1.2 Mg_2_SO_4_, 11.1 glucose, and 1.6 CaCl_2_.

All salts were purchased from Sigma Aldrich (Schnelldorf, Germany). XE991 was purchased from Ascent Scientific (Bristol, UK) or Tocris (Bristol, UK). 4-aminopyridine (4-AP), glibenclamide, l-cysteine, 4-propargylglycine (PPG), ß-cyano-l-alanine (BCA) and NaHS were from Sigma–Aldrich (Schnelldorf, Germany). 2-(aminooxy)-acetic acid (AOAA) was purchased from Tianjin Dihao Chemicals Co., Ltd. (Tianjin, China). 5-(4-hydroxyphenyl)-3H-1,2-dithiole-3-thione (ADTOH) was generously donated by Prof. Dr. Tsikas, Medical School Hannover, Department of Clinical Pharmacology, Hannover, Germany.

All values are given as mean + SEM. Paired and unpaired Student’s t-tests or ANOVA were used as appropriate. A value of p less than 0.05 was considered statistically significant; n represents the number of arteries tested. Local authorities approved the studies according to established guidelines.

## Results

### Contraction of Rat Aortic Rings with and without Perivascular Fat Under CSE or KCNQ Channel Inhibition

**Figure 1:**
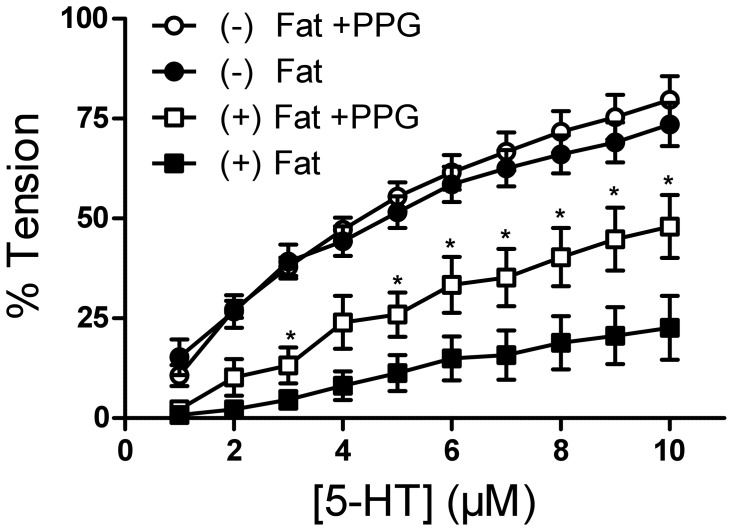
Serotonin (5-HT) dose response curves of rat aortic rings with (Fat (+)) and without (Fat (−)) perivascular fat with and without preincubation with PPG (30 min, 10 mmol/l) in Mulvany Small Vessel Myograph. * p<0.05. n = 7 in (−) Fat rings. n = 6 in (+) Fat rings.

We first investigated the role of CSE-dependent H_2_S in the anti-contractile effect of perivascular fat (PVAT) in rat aorta. Rat aortic rings without perivascular fat ((−) Fat) showed a significantly stronger contraction in response to 1–10 µmol/l serotonin than vessels with ((+) Fat) perivascular fat. Incubation with CSE inhibitor PPG (10 mmol/l, 30 min) inhibited the anti-contractile effect of (+) Fat in rat aortic rings in Mulvany myograph system (Figure 1). PPG did not affect contraction in (−) Fat aortic rings. Similar results were obtained in CSE inhibition with 5 mmol/l BCA or 10 mmol/l PPG in Schuler Tissue Bath System [25]. The CBS inhibitor AOAA (1 mmol/l, 30 min) did not affect the anti-contractile effects of perivascualr fat (Figure S1). These data indicate that CSE-H_2_S is involved in the anti-contractile effects of (+) Fat in rat aortas.

**Figure 2:**
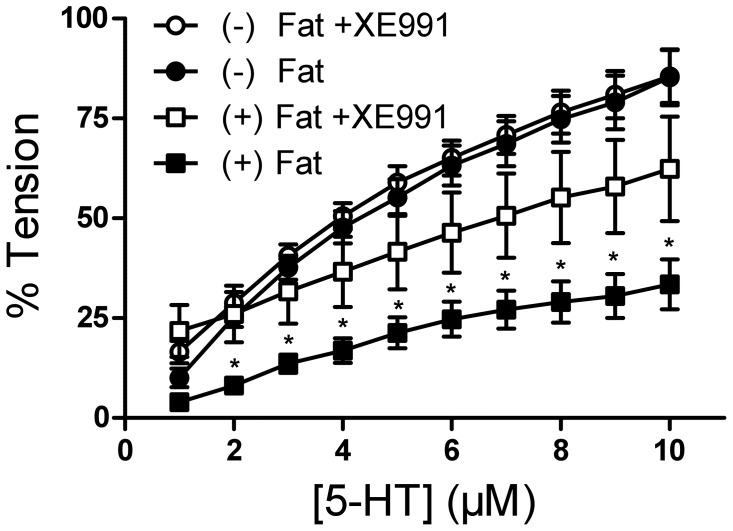
Serotonin (5-HT) dose response curves of rat aortic rings with ((+) Fat) and without ((−) Fat) perivascular fat with and without preincubation with XE991 (15 min, 30 µmol/l) in Mulvany Small Vessel Myograph. * p<0.05. n = 7 for all groups.

We next examined the contractile response of rat aortas to serotonin under KCNQ channel inhibition with XE991. Incubation with XE991 (30 µmol/l) did not modify the dose response curve for serotonin in (−) Fat rat aortic rings. However, it reduced the anti-contractile effects of (+) Fat in rat aortic rings in Mulvany myograph system (Figure 2). Similar results were obtained in Schuler Tissue Bath System [25]. The K_v_ channel inhibitor 4-aminopyridine (4-AP, 2 mM) produced similar effects [21], [25]. These data suggest that XE991-sensitive KCNQ-type K_v_ channels mediate at least in part the ADRF effects in rat aortas.

### Relaxation of Rat Aortic Rings by NaHS Under XE991, 4-AP, and Glibenclamide

**Figure 3:**
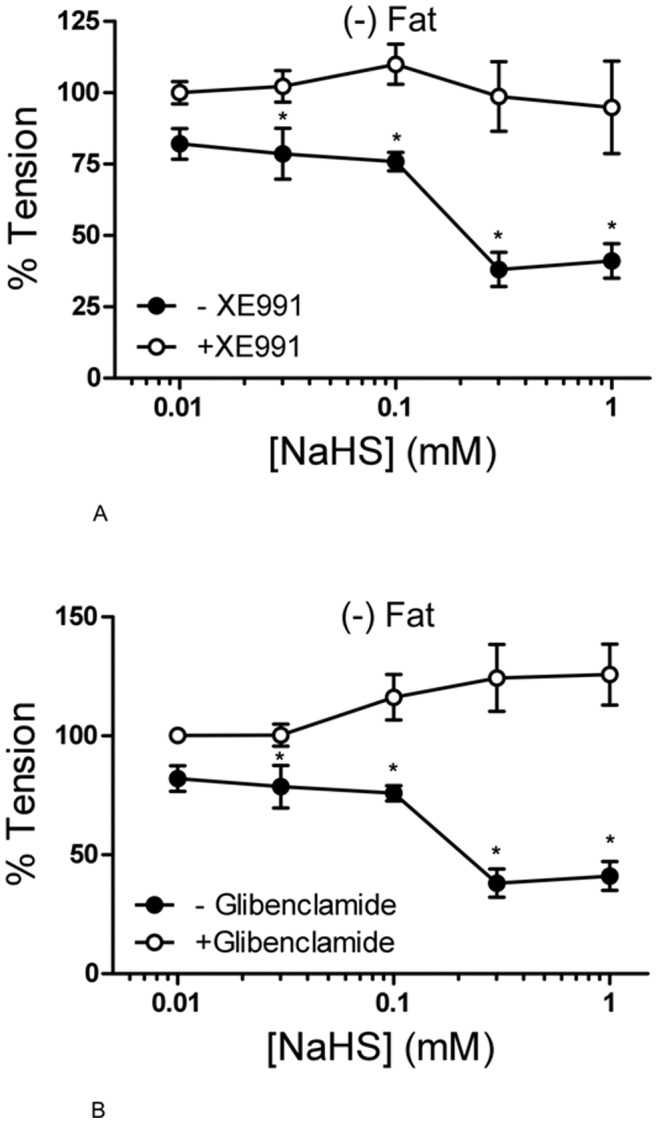
NaHS dose response curve of rat aortic rings without perivascular fat with and without preincubation with XE991 (15 min, 30 µmol/l) (panel A) or with and without preincubation with glibenclamide (15 min, 3 µmol/l) (panel B). Rat aortic rings were precontracted with 5-HT (2 µmol/l) and then stepwise relaxed with NaHS (0.01–1 mmol/l). * p<0,05. n = 9 in control groups. n = 6 in groups with XE991 and glibenclamide, respectively.

**Figure 4:**
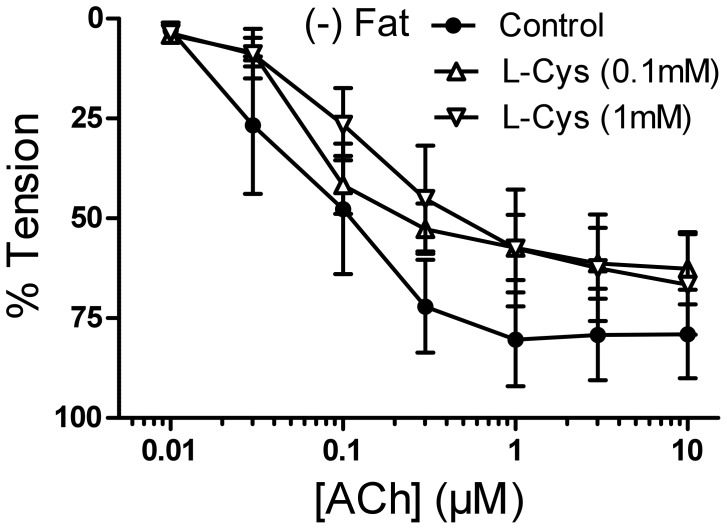
L-cysteine does neither produce vasorelaxation of rat aortic rings without perivascular fat nor affect endothelium-dependent vasorelaxation by acetylcholine (100 µmol/l and 1 mmol/l L-cysteine). All values are p>0.05. n = 6 in each group.

Two different KCNQ channel openers (retigabine and VRX0621688) produced dose-dependent vasorelaxations of rat aortas and these effects were blocked by XE991 [25]. We tested the hypothesis that exogenous H_2_S produced similar effects. One group of vessels was pre-incubated with 30 µmol/l XE991 (15 min) before application of 1 µmol/l serotonin. Two other groups of vessels were either preincubated with the K_v_ channel inhibitor 4-AP (2 mmol/l, 10 min) or with the K_ATP_ channel inhibitor glibenclamide (3 µmol/l, 15 min). NaHS induced strong vasorelaxation in serotonin pre-contracted (−) Fat rat aortic rings. This relaxation reversed by both XE991 (Figure 3A) and 4-AP (data not shown). Of note, glibenclamide also attenuated the NaHS-induced relaxation (Figure 3B), which is consistent with previous data [11], [13], [38]. Taken together, the present results indicate that exogenous H_2_S can induce a strong relaxation in pre-contracted rat aortic rings mediated by opening of KCNQ-type K_v_ channels and K_ATP_ channels, possibly in a cooperative, non-additive manner. L-cysteine (<1 mmol/l) did not produce relaxation of (−) Fat rat aortic rings nor affected endothelium-dependent relaxation by acetylcholine (Figure 4), indicating that endothelial H_2_S production is unlikely involved.

### Contraction of Mouse Aortic Rings with and without Perivascular Fat Under CSE and CBS Inhibition

**Figure 5:**
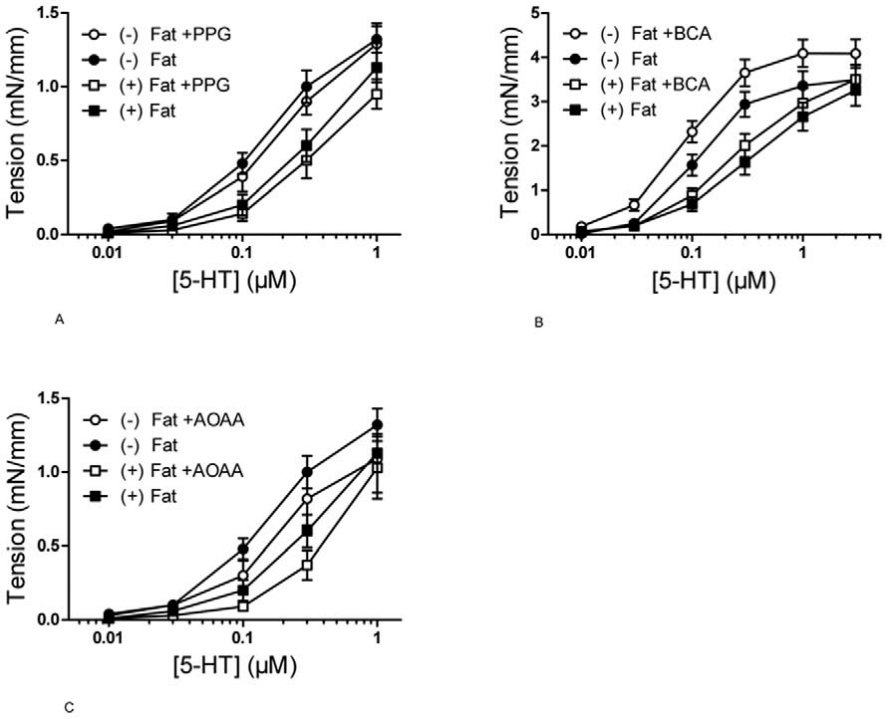
Effects of CSE and CBS inhibitors on serotonin (5-HT) dose response curve of mouse aortic rings with and without perivascular fat. Mouse aortic rings were either non-treated or preincubated with PPG (30 min, 1–10 mmol/l) (panel A), BCA (30 min, 5 mmol/l) (panel B) or AOAA (30 min, 1 mmol/l) (panel B), and then stepwise contracted with 5-HT (0.01–1 µmol/l).) * p<0,05. n = 21 in groups without preincubation. n = 14 for groups with PPG, n = 8−9 for groups with BCA and n = 5 for groups with AOAA.

We next constructed dose-response curves for serotonin in mouse aortas with and without PVAT. All experiments on mouse aortas were performed in Mulvany myograph system. Two groups of vessels were incubated with the CSE inhibitors PPG (1–10 mmol/l, 30 min) or BCA (5 mmol/l, 30 min), another group of vessels was incubated with the CBS inhibitor AOAA (1 mmol/l, 30 min). Neither treatment influenced the serotonin dose-response curves of (+) Fat and (−) Fat mouse aortic rings (Figures 5A, B and C). These data indicate that neither CSE- nor CBS-derived H_2_S has a role in the anti-contractile effect of perivascular fat in mouse aortas.

### Contraction of Mouse Aortic Rings with and without Perivascular Fat Under KCNQ Channel Inhibition

**Figure 6:**
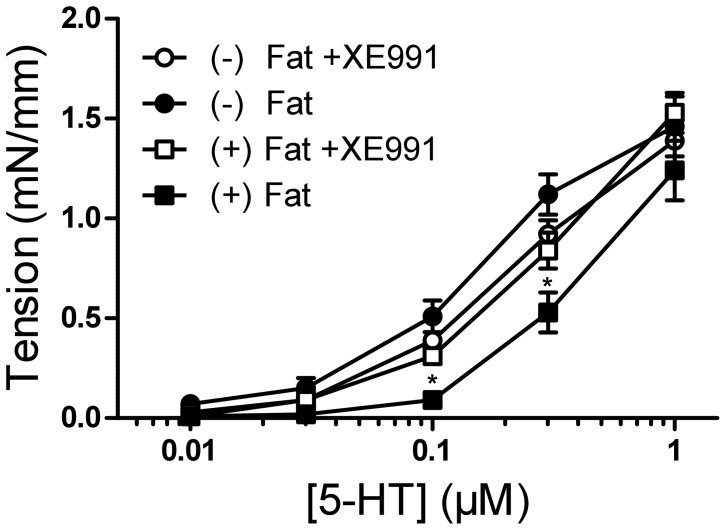
Effects of XE991 on serotonin (5-HT) dose response curve of mouse aortic rings with ((+) Fat) and without ((−) Fat) perivascular fat. Mouse aortic rings were either non-treated or preincubated with XE991 (15 min, 30 µmol/l) and then stepwise contracted with 5-HT (0.01–1 µmol/l). * p<0,05. n = 10 in groups without preincubation. n = 6 for groups with XE991.

We then tested whether KCNQ channel inhibition with 30 µmol/l XE991 impacted the contractile response of mouse aortic rings induced by 0.01–1 µmol/l serotonin. Dose-response curves of (+) Fat mouse aortic rings with XE991 pretreatment were significantly shifted to the left whereas there was no difference in the contractile response in (−) Fat mouse aortic rings (Figure 6). These results provide indirect evidence that XE991-sensitive KCNQ-type K_v_ channels are involved in the anti-contractile effects of PVAT in mouse aortic rings.

### Relaxation of Mouse Aortic Rings by Exogenous H_2_S

**Figure 7:**
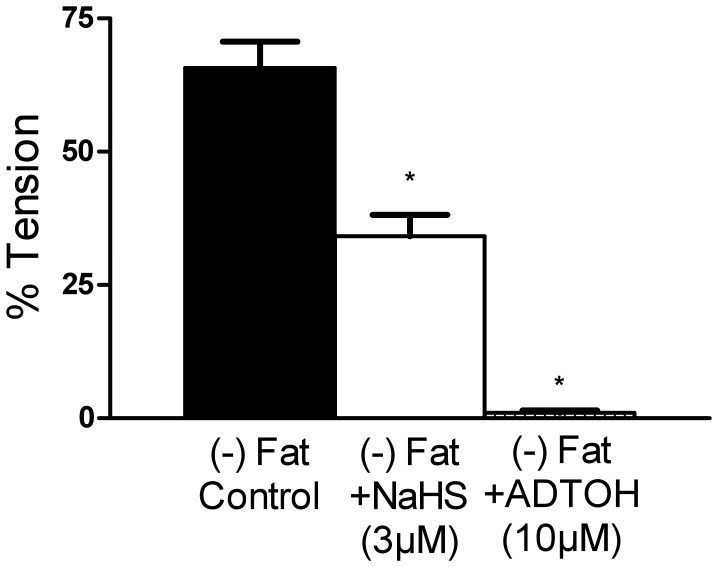
NaHS and ADTOH induced relaxations of (−) Fat mouse aortas. Mouse aortic rings were precontracted with 5-HT (1 µmol/l). Tension was recorded after 30 min. * p<0.05. n = 16 in (−) Fat control. n = 11 in NaHS group. n = 8 in ADTOH group.

In a following set of experiments, we investigated whether exogenous H_2_S can induce long-lasting relaxations in mouse aortic rings. Serotonin precontracted (−) Fat aortas were stepwise treated with either 0.01–3 mmol/l NaHS or 0.01–100 µmol/l ADTOH. Control groups were non-treated, with 1 µmol/l serotonin precontracted mouse aortic rings with and without perivascular fat. (−) Fat aortic rings without treatment showed a spontaneous relaxation by 34.4%. Both H_2_S donors induced a strong dose-dependent relaxation of pre-contracted (−) Fat mouse aortic rings. NaHS induced a relaxation by 65.82%, ADTOH by 98.96% (Figure 7), suggesting that exogenous H_2_S is able to produce sustained relaxation of mouse aortic rings.

### Relaxation of Mouse Aortic Rings by Exogenous H_2_S Under KCNQ Channel Inhibition

**Figure 8:**
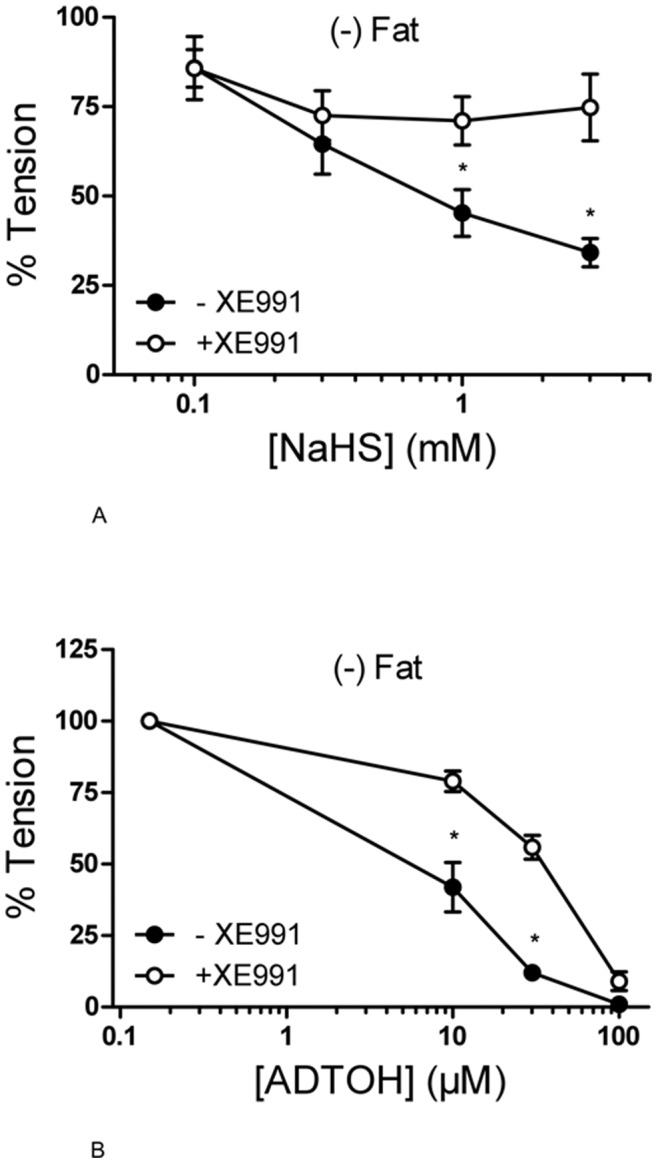
Effects of XE991 (15 min, 30 µmol/l) on NaHS (panel A) and ADTOH (panel B) relaxation of mouse aortic rings without ((−) Fat) perivascular fat. Mouse aortic rings were precontracted with 5-HT (1 µmol/l), and then stepwise relaxed with either NaHS (0.01–1 mmol/l) or ADTOH (10–100 µmol/l). * p<0,05. n = 8 in NaHS control. n = 9 in NaHS group with XE991. n = 6 in both ADTOH groups.

To test the hypotheses that KCNQ channel inhibition by XE991 influences the NaHS- and ADTOH- induced relaxation, we produced dose-response curves of (−) Fat mouse aortic rings to either agent with and without preincubation of XE991 (30 µmol/l, 15 min). Incubation with XE991 inhibited NaHS-induced relaxation of (−) Fat mouse aortic rings (Figure 8A). The dose-response curve to ADTOH was shifted to the right in (−) Fat vessels pretreated with XE991 (Figure 8B). These data indicate that the vasorelaxation by exogenous H_2_S is at least in part mediated by KCNQ channel activation.

### Relaxation of Mouse Aortic Rings by l-cysteine with and without CSE Inhibition

**Figure 9:**
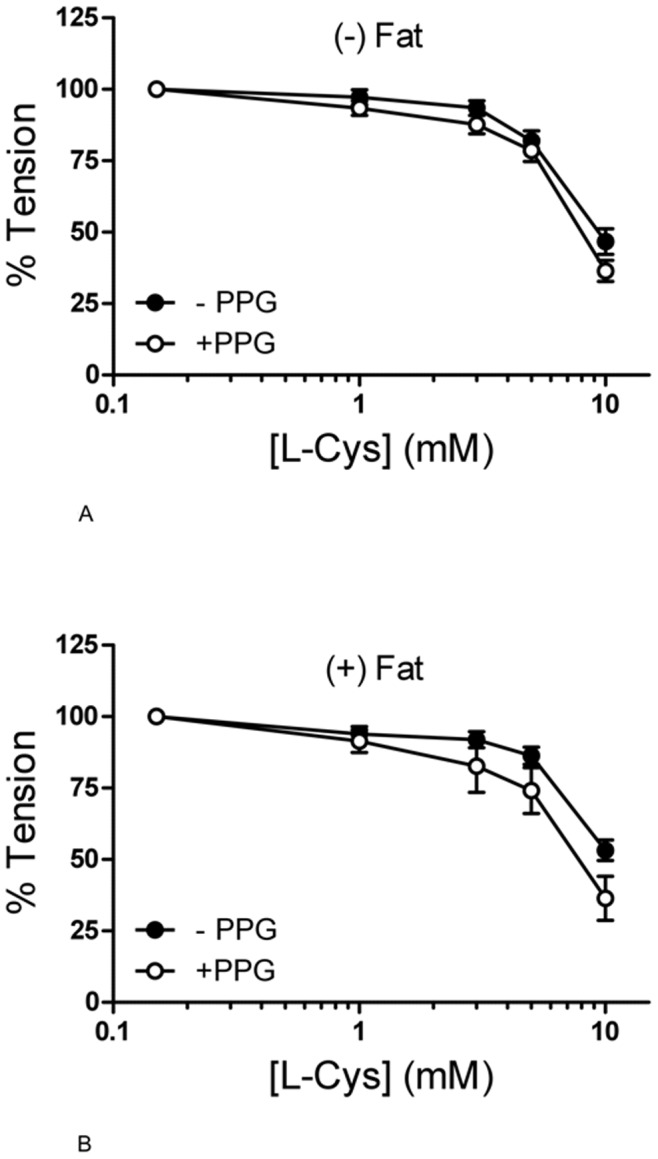
L-cysteine dose response curves of mouse aortic rings without perivascular fat (panel A) and with perivascular fat (panel B) with and without preincubation with PPG (30 min, 10 mmol/l). p>0.05 for all values. n = 11 in l-cysteine (−) fat control. n = 5 in (−) fat group with PPG. n = 7 in l-cysteine (+) fat control. n = 6 in (+) fat group with PPG.

To investigate the role of endogenous H_2_S as a potent vasorelaxant, we produced dose response curves of pre-contracted (−) Fat mouse aortic rings to l-cysteine. To further study the role of CSE in this potential vasorelaxation one group of vessels was pre-treated with PPG (10 mmol/l, 30 min). L-cysteine produced a dose-dependent relaxation of (−) Fat and (+) Fat mouse aortic rings. CSE inhibition with PPG was without effect (Figure 9A and B).

### Vascular Expression of CSE

(+) Fat rat aortic rings showed marked expression of CSE, whereas CSE expression was non-detectable low in rat aortic rings without perivascular fat ((−) Fat) (Figure S2). A similar expression pattern has been observed in mouse aortas, although expression in (+) Fat tissue seemed to be lower compared to (+) Fat tissue of rats (Figure S2). These data indicate that CSE is predominantly expressed in aortic PVAT of both rats and mice, with possibly higher expression in PVAT of rats compared to mice.

## Discussion

**Figure 10:**
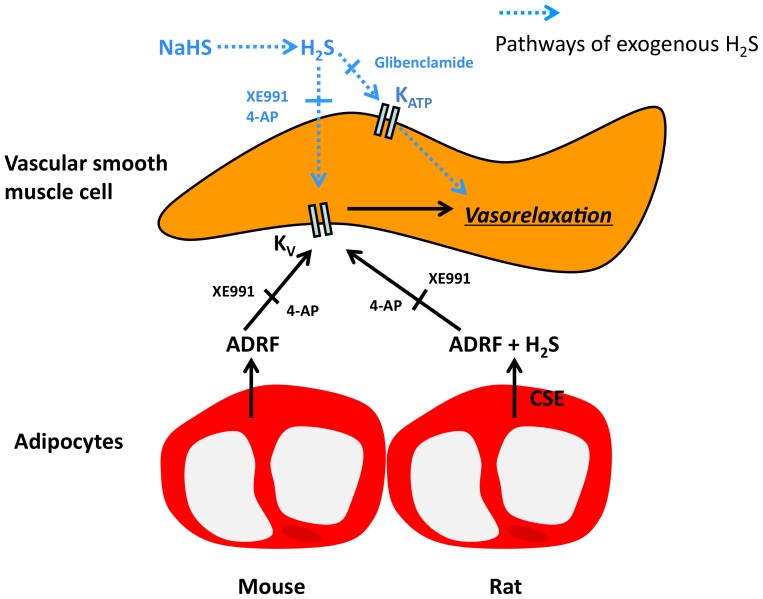
Schematic illustration on differential effects of cystathionine-γ-lyase (CSE)–dependent vasodilatory H_2_S in periadventitial vasoregulation of rat and mouse aortas. 4-AP, 4-aminopyridine.

With our study, we provide novel insights into the complex interaction between ADRF and H_2_S as paracrine mediators of vascular tone. First, H_2_S modulates vascular tone of rat aortas in a PVAT-dependent manner. We found that CSE inhibition strongly inhibits the anti-contractile effect of perivascular fat in rat aortas, which is similar to previous findings [25], [33]. Furthermore, we showed that exogenous H_2_S induces vasorelaxation in rat aortas and this effect can be blocked by inhibition of KCNQ-type K_v_ channels and K_ATP_ channels. Thus, H_2_S might act as an ADRF in rat aortas, which supports previous data [33], [39]. Figure 10 is a signal transduction scheme depicting the assumed pathways.

Secondly, our data question that CSE-H_2_S is a primary ADRF candidate in mouse aortas. We showed that CSE inhibition with PPG in mouse aortas did not influence the anti-contractile effects of perivascular fat, which is in contrast to our findings on rat aortas (Figure 10). CBS is another enzyme catalyzing the formation of H_2_S [1], [40]. Although CBS is like CSE able to produce endogenous H_2_S from l-cysteine, this enzyme does not play a role in the regulation of vascular tone by endothelial and intrinsic smooth muscle mechanisms (for review see [1]). Our study is an extension of these studies. We found that inhibition of CBS does not influence the ADRF effect in mouse aortas. However, exogenous H_2_S was able to induce a strong vasorelaxation in this set of experiments. Similar to rat aortas, this effect was blocked by KCNQ-type K_v_ channel inhibition with XE991 (Figure 10). Furthermore, exogenous l-cysteine, the precursor of endogenously produced H_2_S, was able to induce relaxation in mouse aortas. However, this effect was not blocked by CSE inhibition and independent on the presence of PVAT. Together, the present results suggest that endogenous H_2_S generated by CSE (or CBS) is not involved in the anti-contractile effects of PVAT in mouse aortas. However, our data do not exclude the possibility that H_2_S generated from external, non-vascular sources, such as the endothelial 3-mercaptopyruvate-sulfurtransferase (3MST) [4] or derived from red blood cells [41], might modulate vascular tone of mouse aortas (Figure 10). Further studies are needed to test this hypothesis. Nevertheless, our study shows that H_2_S acts rather as modulator of ADRF than primary ADRF in mouse aortas and possibly other arteries. Inhibitors of 3MST are not yet available to test this hypothesis. Future genetic approaches could facilitate our understanding of the relationships between endogenous H_2_S production, PVAT and vascular tone.

Thirdly, our study demonstrates that KCNQ-type K_v_ channel opening is a powerful mechanism to induce relaxation in rat and mouse systemic arteries. Both ADRF and H_2_S can utilize this mechanism to produce vasorelaxation. Inhibition of XE991-sensitive KCNQ-type K_v_ channels strongly attenuated the anti-contractile effect of perivascular fat in both rat and mouse aortas, suggesting that there is a common mechanism of ADRF to act *via* opening of these channels. Also, H_2_S-induced relaxation in rat and mouse aortas was almost completely inhibited by XE991 (Figure 10). Thus, KCNQ channel opening might serve as an important mechanism to induce relaxation in hypertension, diabetes and obesity associated with ADRF malfunction, and might represent a promising novel target for the development of new therapeutic strategies in cardiovascular diseases.

CSE-derived H_2_S has been previously proposed as direct ADRF candidate in rat aortas [25], [33]. Of note, there are obviously species differences in the CSE expression in the vasculature. Fang et al. identified significant CSE expression in periadventitial adipocytes and smooth muscle cells but not in the endothelium of rat aortas [33], whereas Yang et al. observed CSE expression in the endothelium but not in smooth muscle cells of mouse aortas [5]. However, the expression of CSE in PVAT of mice had not been studied so far. Nevertheless, our functional studies indicate that CSE-H_2_S is not involved in the anti-contractile effects of PVAT in mouse aortas. These data support the notion that, at least in mouse aorta, CSE-H_2_S does not act as a primary ADRF, instead it is probably an ADRF modulator. Future biochemical studies should investigate putative CSE expression in periadventitial adipocytes over a broad range of different vessels and species. Although PPG and BCA are commonly used as inhibitors of H_2_S biosynthesis, they have low potency and selectivity, and limited cell-membrane permeability [1]. When interpreting the data, the severe limitations of these experimental compounds and potential off-target effects must be kept in mind. Thus, future genetic approaches are needed to investigate the complex interaction between perivascular fat and hydrogen sulfide. CSE deficient mice have been recently engineered [5], [40] and may help to clarify the situation in future studies.

We confirmed that H_2_S can relax systemic large arteries of rats and mice [11], [12], [13], [15], [38], [42]. Of note, this effect can be potentiated by application of new H_2_S-releasing hybrids, an observation that might be therapeutically relevant for the treatment of vascular dysfunction. In our studies, NaHS (3 mmol/l) produced a relaxation of over 60%. However, ADTOH (100 µmol/l) induced a relaxation of greater than 95%. ADTOH is a dithiole-3-thione moiety of H_2_S-releasing aspirin. Therefore, ADTOH does not only liberate H_2_S but can also suppress thromboxane A_2_ activity [43]. Previous studies investigated various pro-inflammatory cytokines secreted by PVAT in states of ischemia and hypoxia. The studies identified the involvement of components of the renin angiotensin system, IL-1, IL-6, TNF α, and C-reactive protein [31], [44]. Additional secretion of reactive oxygen species causes oxidative stress which, in combination with inflammation, can potentiate vascular dysfunction [45]. Kotsis et al. showed that malfunction of adipose tissue in obesity is associated with the release of thromboxane A_2_ and other pro-inflammatory molecules [44]. Therefore, our study does not only provide information about H_2_S as a potent ADRF modulator but also presents a novel link between its vasodilatory and anti-inflammatory actions in states of ADRF malfunction. Of note, we previously found that hypertension in spontaneously hypertensive (SHR) rats and New Zealand obese (NZO) mice is associated with ADRF malfunction [37], [46]. Therefore, our study supports that new H_2_S-releasing hybrids, such as ADTOH, are novel and exciting drugs to intervene in the progression of vascular dysfunction in cardiovascular diseases and hypertension that warrant further investigation.

This study investigated the complex interaction between perivascular fat and H_2_S as regulator of vascular tone and modulator of inflammation in large arteries. We found that CSE-derived H_2_S is rather an ADRF modulator than a direct candidate for ADRF in mouse aortas. We identified KCNQ channel opening as a powerful mechanism to induce relaxation in H_2_S-dependent vasoregulation. Furthermore, we identified ADTOH as a putative molecule to intervene in CSE/H_2_S-dependent vasoregulation, which may open new therapeutic options in the treatment of periadventitial vascular dysfunction. Further studies are needed to investigate the putative pharmacological potential of ADTOH and KCNQ channel opening in these scenarios. Our study is limited by utilizing pharmacological approaches to reveal a role of CSE in periadventitial vasoregulation by perivascular adipose tissue. Genetic approaches are necessary to definitely clarify the role of CSE-H_2_S in this regulation. CSE gene-deficient mice are available and should be used for this purpose in future studies.

## Supporting Information

Figure S1 **Western blot analysis of CSE levels in mouse and rat aortic rings with ((+) Fat) and without ((−) Fat) perivascular fat.**
(TIF)

Figure S2 **Effects of CBS inhibitor AOAA on serotonin (5-HT) dose response curve of rat aortic rings with and without perivascular fat.** Rat aortic rings were either non-treated or preincubated with AOAA (30 min, 1 mmol/l), and then stepwise contracted with 5-HT (0.01–1 µmol/l).) * p<0.05. n = 10 in each group.
(TIF)
