# Supplementary figures and images for: Differential Effects of Cystathionine-γ-lyase–Dependent Vasodilatory H2S in Periadventitial Vasoregulation of Rat and Mouse Aortas
Source: PLoS One. 2012 Aug 3;7(8):e41951. doi: 10.1371/journal.pone.0041951 (PMC3411702; doi:10.1371/journal.pone.0041951)

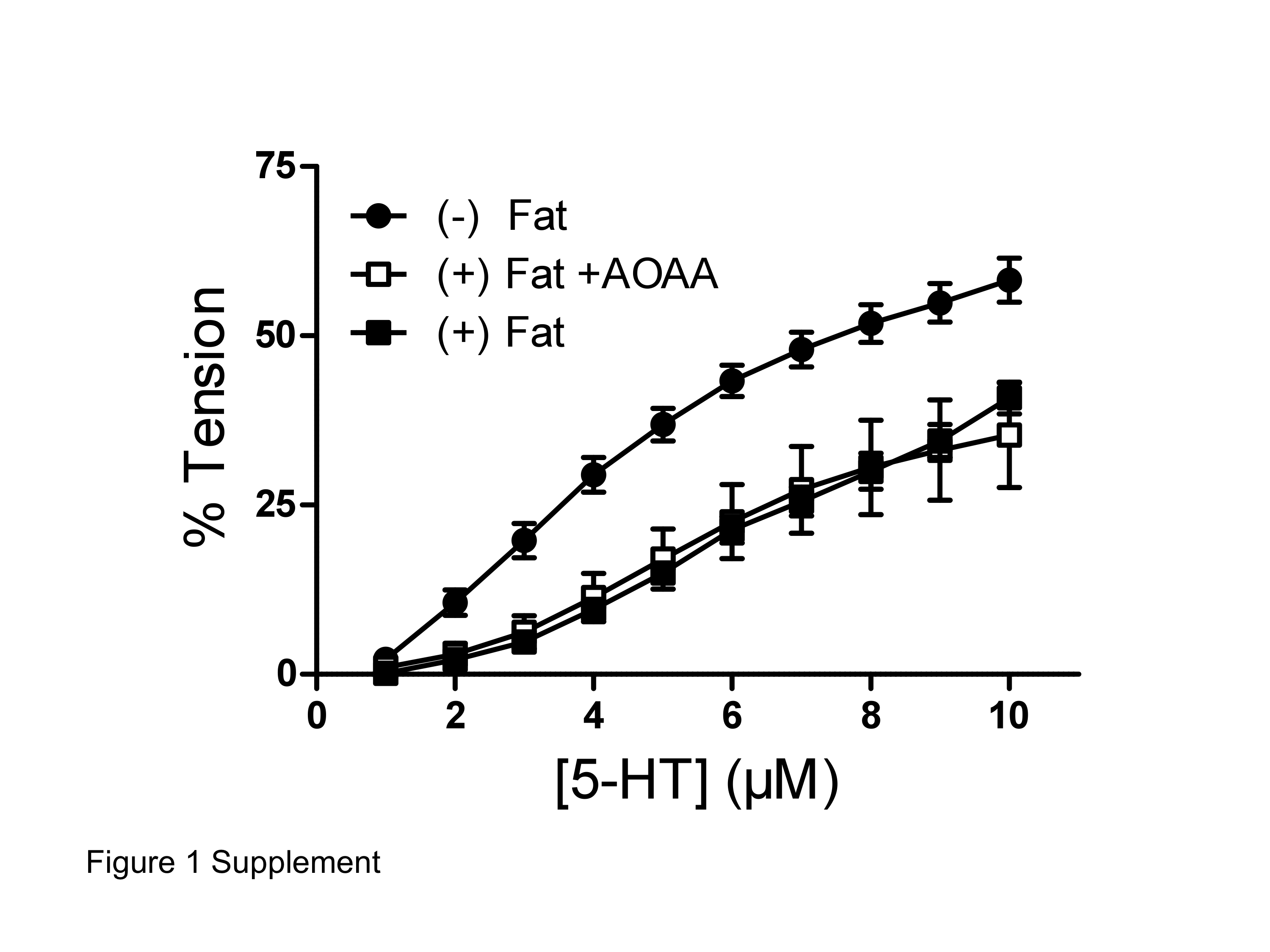

Supplement: Figure S1 — Western blot analysis of CSE levels in mouse and rat aortic rings with ((+) Fat) and without ((−) Fat) perivascular fat. (TIF) [file pone.0041951.s001.tif]

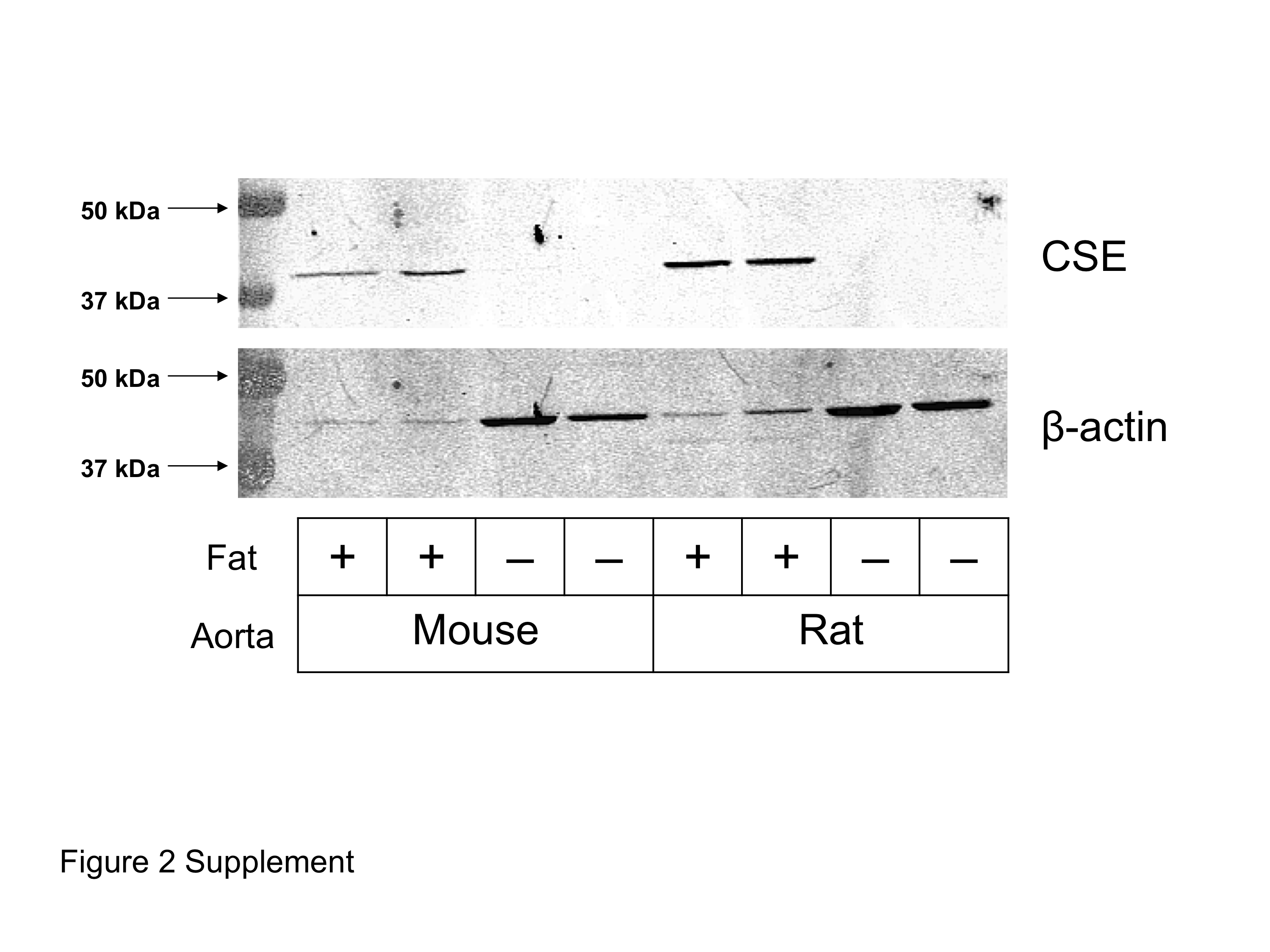

Supplement: Figure S2 — Effects of CBS inhibitor AOAA on serotonin (5-HT) dose response curve of rat aortic rings with and without perivascular fat. Rat aortic rings were either non-treated or preincubated with AOAA (30 min, 1 mmol/l), and then stepwise contracted with 5-HT (0.01–1 µmol/l).) * p<0.05. n = 10 in each group. (TIF) [file pone.0041951.s002.tif]
